# Impact of Coexisting Pulmonary Diseases on Survival of Patients With Lung Adenocarcinoma: A STROBE-Compliant Article

**DOI:** 10.1097/MD.0000000000000443

**Published:** 2015-01-30

**Authors:** Zhi-Hong Jian, Jing-Yang Huang, Pei-Chieh Ko, Shiou-Rung Jan, Oswald Ndi Nfor, Chia-Chi Lung, Wen-Yuan Ku, Chien-Chang Ho, Hui-Hsien Pan, Yung-Po Liaw

**Affiliations:** From the Department of Public Health and Institute of Public Health (Z-HJ, J-YH, P-CK, S-RJ, ONN, C-CL, W-YK, Y-PL), Chung Shan Medical University; Department of Family and Community Medicine (C-CL, Y-PL), Chung Shan Medical University Hospital, Taichung City; Department of Physical Education (C-CH), Fu Jen Catholic University, New Taipei City; and Department of Pediatrics and School of Medicine (H-HP), Chung Shan Medical University, Taichung City, Taiwan.

## Abstract

Asthma, chronic obstructive pulmonary disease (COPD), and pulmonary tuberculosis (TB) are common pulmonary diseases associated with lung cancer. Besides, smoking is more prevalent in Taiwanese men. This study evaluated gender disparities in coexisting pulmonary diseases on survival of patients with lung adenocarcinoma.

Patients newly diagnosed with lung cancer between 2003 and 2008 were identified from Taiwan National Health Insurance Research Database. Cases with lung adenocarcinoma were further confirmed using the Cancer Registry Database and followed up until the end of 2010. Cox proportional hazard regression was used to calculate the hazard ratio (HR) of coexisting asthma, COPD, and/or TB to estimate all-cause mortality risk.

During the study period, 13,399 cases of lung adenocarcinoma were identified. The HRs of adenocarcinoma in men and women were 1.20 (95% confidence interval [CI], 1.10–1.30) and 1.05 (95% CI, 0.95–1.16), respectively, for individuals with asthma, 1.32 (95% CI, 1.16–1.51) and 0.97 (95% CI, 0.89–1.05), respectively, for COPD, and 0.99 (95% CI, 0.93–1.06) and 1.06 (95% CI, 0.86–1.32), respectively, for individuals with TB. Specifically, among men with coexisting pulmonary diseases, the HRs were 1.63 (95% CI, 1.25–2.13), 1.31 (95% CI, 1.08–1.59), and 1.23 (95% CI, 1.11–1.36) for individuals with asthma + COPD + TB, asthma + COPD, and COPD + TB, respectively. However, there was no increase risk of mortality among women with coexisting pulmonary diseases.

Coexisting pulmonary diseases are at an elevated risk of mortality among male patients with lung adenocarcinoma. Such patients deserve greater attention while undergoing cancer treatment.

## INTRODUCTION

Lung cancer remains the leading cause of cancer death worldwide with adenocarcinoma being the most common histologic type.^1,2^ Lung cancer survival mainly depends on patients’ characteristics, gender, histologic cell types, stage, and comorbidities.^3–6^ With the increasing mean age, there is increased probability of comorbidities in patients with lung cancer. Tammemagi et al^7^ found that more than half of patients with lung cancer had ≥3 comorbidities. However, gender differences in comorbidities of patients with lung cancer were not reported.

However, studies on impact of specific comorbidities on lung cancer survival are limited and have yielded conflicting results, and the sample size was small. Tammemagi et al^7^ found 19 of 56 comorbidities to independently predict of lung cancer survival in a cohort of 1155 patients. Battafarano et al^8^ investigated the survival impact of comorbidity in 451 patients with resected stage I nonsmall cell lung cancer and used the Kaplan–Feinstein index as an aggregate measure of comorbidity, rather than reporting the results for specific comorbidities. Sekine et al^9^ found that lung cancer patients with chronic obstructive pulmonary disease (COPD) had poor long-term survival and high incidence of tumor recurrence in a retrospective chart review of 442 patients with stage IA lung cancer after complete resection. Brown et al^10^ (the Second National Health and Nutrition Examination Survey Mortality Study) analyzed 196 patients who died of lung cancer and found that asthma increased risk of lung cancer mortality in nonsmokers. The survival of patients with coexisting pulmonary tuberculosis (TB) and lung cancer remains controversial.^3,11^

The burden of coexisting pulmonary comorbidities and the impacts on survival of specific types of lung cancer have greatly been underestimated. It is an important public health issue in Taiwan where the prevalence of asthma (11.9%), COPD (2.48%), and TB is high.^12,13^ A total of 57,405 new cases of TB were identified in Taiwan between 2005 and 2007.^14^ It is hypothesized that inflammation may initiate or promote carcinogenesis in the lung.^15^ Among common pulmonary diseases with inflammation, asthma,^16^ COPD,^17^ and TB^18^ have been associated with the development of lung cancer. Furthermore, the prevalence of smoking in Taiwan population is reported at 45.7% in men and 4.8% in women.^19^ This can drive the observed differences in survival between men and women. Smoking has been shown to influence lung cancer survival independent of comorbidities.^20^ The objective of this study was to evaluate the impact of coexisting pulmonary diseases (asthma, COPD, and/or TB) on survival by gender for patients with lung adenocarcinoma.

## METHODS

### Data Source

This retrospective cohort study was conducted using data obtained from Taiwan's National Health Insurance Research Database (NHIRD), Taiwan Cancer Registry Database (TCRD), and National Death Registry Database (NDRD). Taiwan's National Health Insurance enrolls >99% of Taiwan's 23 million residents in Taiwan. The NHIRD contains comprehensive health care information, including diagnoses, prescriptions, and information on ambulatory and inpatient care. The NHIRD is one of the largest datasets in the world and numerous researches using NHIRD have been published.^21–23^ The data from NHIRD were used to measure patients’ comorbidities. This study was approved by the Institutional Review Board, Chung Shan Medical University Hospital, Taichung, Taiwan.

### Patient Identification

Individuals aged ≥20 years who were free of lung cancer before 2002 were identified from the NHIRD. Individuals with incomplete information, such as sex and registry data, were excluded. Cases newly diagnosed with lung cancer were retrieved between 2003 and 2008, and were followed up until death, loss of follow-up, or the end of 2010. Lung cancer was identified using the International Classification of Diseases, Ninth Revision, Clinical Modification (ICD-9-CM) code 162.

The adenocarcinoma types of lung cancer were further confirmed by TCRD. All major cancer care hospitals in Taiwan are obligated to submit cancer type, initial tumor stages, and histology to the Taiwan Cancer Registry established by the Bureau of Health Promotion, Department of Health. Lung cancers were coded by ICD-9-CM 162 or ICD 10 C34.0–C34.3, C34.8, and C34.9 in TCRD. Morphological diagnoses were coded using the ninth revision of the International Classification of Diseases for Oncology (ICD-O), primarily based on ICD-O codes 80503, 81402, 81403, 81413, 81433, 82113, 82503, 82513, 82523, 82553, 82603, 83103, 83233, 84603, 84803, 84813, 84903, and 85003 for adenocarcinoma.

The NDRD, causes of death classified by ICD-9 CM, was then linked to the NHIRD and TCRD to assess the age of onset of cancer more accurately, estimate person-months follow-up, confirm death and survival time, and reduce potentially unconﬁrmed cancer diagnoses. Survival data were used to summarize the estimated risks of pulmonary diseases for all-cause mortality in patients with lung adenocarcinoma.

### Exposed Variables

The demographics and comorbidities were obtained from the NHIRD. The registration date of lung cancer was defined as the index date. To enhance the reliability of temporal relationship between comorbidities and all-cause mortality of lung adenocarcinoma, cases of asthma, COPD, TB, and other comorbidities diagnosed 2 years before the index date were included. The diagnoses of pulmonary diseases and other comorbidities were confirmed by at least 2 outpatient visits or 1 admission in a year. Baseline pulmonary diseases and other comorbidities were listed as follows: asthma (ICD-9-CM: 493), COPD (ICD-9-CM: 490–492, 494, 496), TB (ICD-9-CM: 010–012, 137.0), chronic renal disease (ICD-9-CM: 585, 586), type II diabetes mellitus (DM) (ICD-9-CM: 250, excluding type 1 DM), hyperlipidemia (ICD-9-CM: 272), and smoking-related cancers (ICD-9-CM: 140–150, 157, 160, 161, 189).

### Statistical Analysis

All data management was conducted using SAS 9.3 software (SAS Institute, Cary, NC). The number of person-months of follow-up was calculated from the time of entry into the study until death or the termination of the study. All-cause mortality rates per 100 person-months were calculated. Univariate Poisson regression was made in order to determine all-cause mortality rate ratios for independent variables, including asthma, COPD, TB, sex, age of lung adenocarcinoma diagnosed, cancer stage, surgery, low income, comorbidities, geographical area, and urbanization. Multivariable analyses were carried out with Cox proportional hazards models to determine the strength of pulmonary diseases and survival of lung adenocarcinoma. In order to evaluate the effects of coexisting pulmonary diseases, 3 separate models were estimated for both genders and adjusted for confounders: a model containing 3 pulmonary diseases, a model containing pulmonary disease combinations, and a count of pulmonary disease model. All comparisons with a *P* value <0.05 were considered statistically significant.

## RESULTS

Between 2003 and 2008, a total of 13,399 patients were diagnosed with lung adenocarcinoma. Demographic characteristics and comorbidities of the study population are listed in Table 1. Patients with asthma, COPD, and TB were at greater risk of all-cause mortality of adenocarcinoma. Their rate ratios were 1.133 (95% confidence interval [CI], 1.131–1.135), 1.059 (95% CI, 1.058–1.060), and 1.172 (95% CI, 1.166–1.179), respectively. Compared with women, there was increased risk of mortality in men with lung adenocarcinoma (rate ratio, 1.459; 95% CI, 1.458–1.460).

**TABLE 1:**
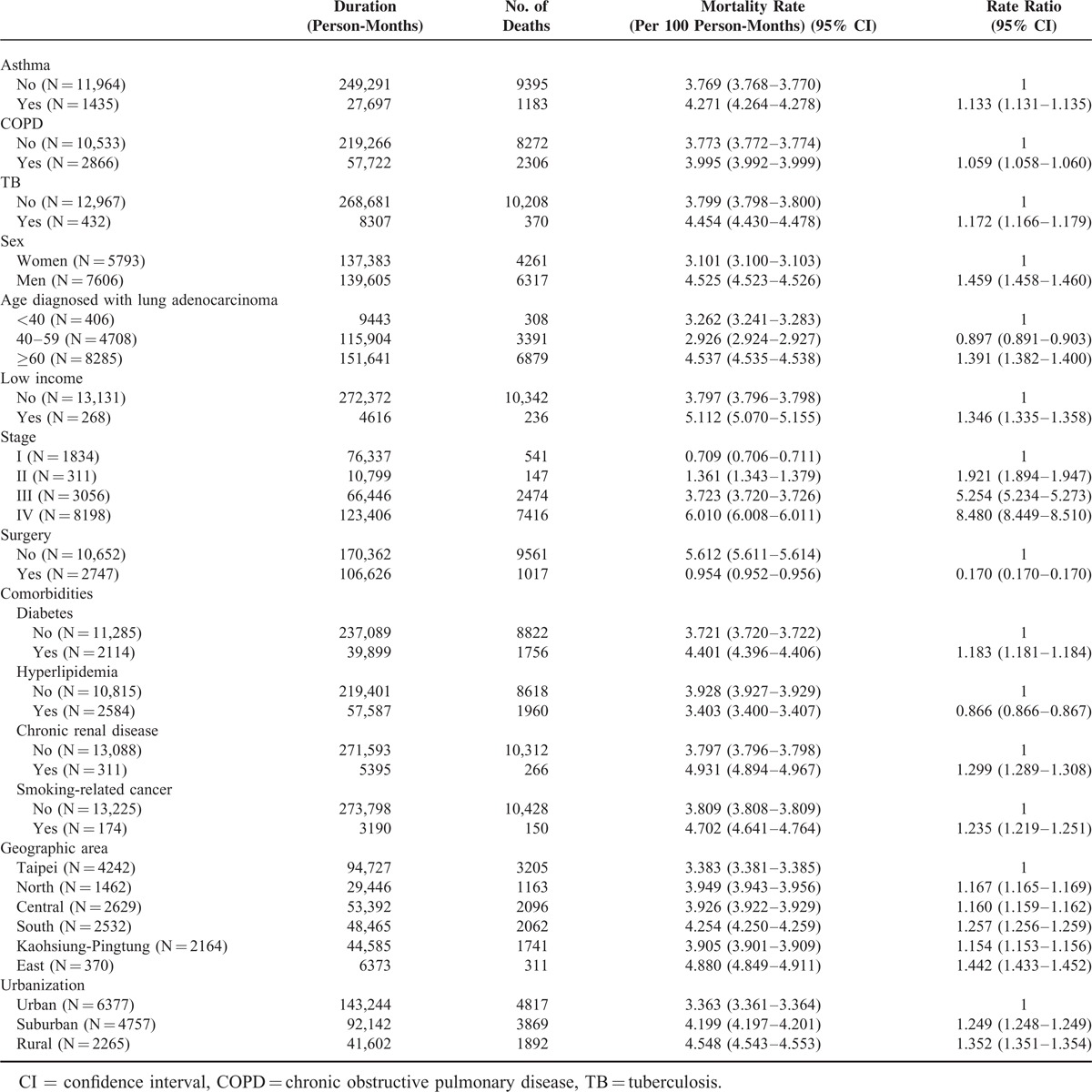
Characteristics of Patients With Lung Adenocarcinoma and All-Cause Mortality Rate, Taiwan, 2003–2010

In Table 2, Cox regression analysis showed that the risk of all-cause mortality was significantly higher in lung adenocarcinoma patients with asthma (hazard ratio [HR], 1.15; 95% CI, 1.08–1.22) and TB (HR, 1.13; 95% CI, 1.01–1.25) but lower in patients with hyperlipidemia (HR, 0.85; 95% CI, 0.81–0.90). There was no increased risk of mortality among patients with COPD (HR, 1.02; 95% CI, 0.97–1.07).

**TABLE 2:**
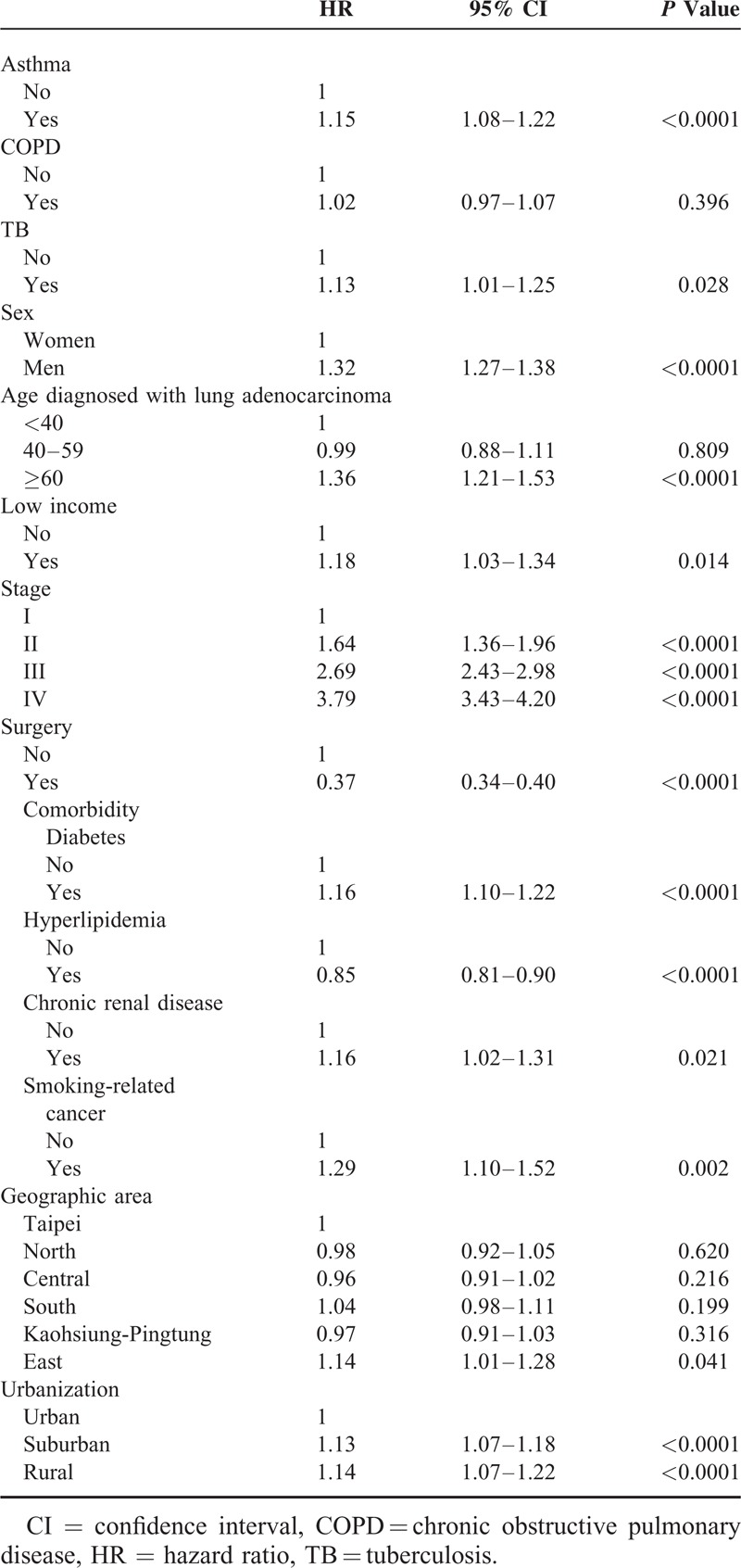
Cox Proportional Model to Estimate the HRs of All-Cause Mortality in Patients With Lung Adenocarcinoma Between 2003 and 2010

Table 3 illustrates the coexisting pulmonary diseases and the risk of all-cause mortality of lung adenocarcinoma according to models and gender after adjusting for age, low income, stage, surgery, comorbidities, geographic area, and urbanization. In model 1, the HRs of pulmonary diseases were higher among male patients with asthma (HR, 1.20; 95% CI, 1.10–1.30) and COPD (HR, 1.32; 95% CI, 1.16–1.51). Among female patients, there was no significant association between mortality and pulmonary diseases. In model 2, the HRs of different combinations of pulmonary diseases were higher among male patients with asthma + COPD + TB (HR, 1.63; 95% CI, 1.25–2.13), asthma + COPD (HR, 1.31; 95% CI, 1.08–1.59), and COPD + TB (HR, 1.23; 95% CI, 1.11–1.36). There was no significant association between survival and combinations of pulmonary diseases among female patients. In model 3, the total number of pulmonary diseases per individual was evaluated and was referred to as the disease count. The counts of pulmonary diseases was shown to increase risk of survival in male patients with any 2 pulmonary diseases (HR, 1.26; 95% CI, 1.15–1.38) and with asthma + COPD + TB (HR, 1.63; 95% CI, 1.25–2.13).

**TABLE 3:**
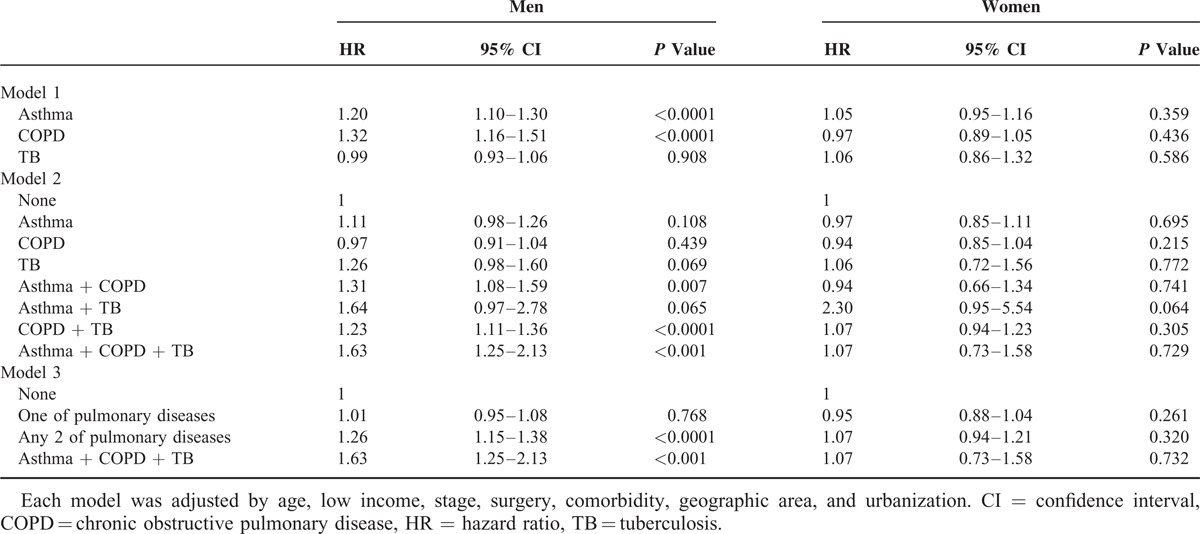
Estimated HRs of All-Cause Mortality Related to Pulmonary Disease Combinations in Patients With Lung Adenocarcinoma by Gender

## DISCUSSION

This study examined how pulmonary diseases are associated with mortality in patients with lung adenocarcinoma. The most important finding in this study is that, unlike specific lung disorders, coexisting pulmonary diseases showed increasing risk of mortality in male patients with lung adenocarcinoma.

Lung cancer mortality increased in patients with coexisting asthma.^10^ COPD has also been associated with worse survival outcomes of lung cancer in men^24^ and a higher incidence of tumor recurrence.^9^ In this study, 3.2% of patients with lung adenocarcinoma had TB. Yu et al^25^ found an increased risk of lung cancer among individuals with TB (HR, 3.32; 95% CI, 2.70–4.09). In a hospital-based study, lung cancer patients with comorbid TB had an increased risk of mortality (HR, 1.30; 95% CI, 1.03–1.65).^26^ In Hong Kong, TB remained an independent predictor of lung cancer death with adjusted HR of 2.81 (95% CI, 1.45–5.42) for nonsmokers and 1.76 (95% CI, 1.13–2.72) for smokers.^11^ Recently, a population-based prospective cohort study indicated that pulmonary TB may be a negative prognostic factor for lung cancer survival (HR, 2.36; 95% CI, 1.13–4.91).^27^ However, Kuo et al^3^ reported in a study of 276 patients with nonsmall cell lung cancer (stages III and IV) that concomitant active TB prolongs survival. In the study by Kuo et al, the sample was therefore not representative of the general lung cancer population. In our study, we analyzed comorbidities that were diagnosed 2 years before the index date. It is less likely that the diagnosis of lung cancer may be delayed mainly because of masking by a tuberculous lesion,^28^ thus influencing the survival.

Coexistence of ≥2 pulmonary diseases had a significantly increased mortality of lung adenocarcinoma. Asthma and COPD may coexist in the same patients. The prevalence rates of asthma–COPD overlap syndrome in Italy were 1.6%, 2.1%, and 4.5% among the 20–44, 45–64, and 65–84 age groups.^29^ Overlap syndrome has been associated with worse lung function, a worse quality of life, more severity and frequency of respiratory exacerbations, and increased mortality and health care utilization than those with asthma or COPD alone.^30–32^ More recently, a cohort study found that history of TB was an independent risk factor for COPD.^33^ Inghammar et al^34^ conducted a population-based cohort study in Sweden in which patients with COPD had about 3-fold increased risk of developing active pulmonary TB. It was also found that patients with COPD who developed active TB had a 2-fold increased risk of death compared with the control subjects with TB. Asthma, COPD, and TB primarily affect the lungs and are the major causes of morbidity and mortality worldwide. Biologically, the additive effects may be explained by compromised immune clearance of *Mycobacterium tuberculosis* and chronic inflammatory processes of the lung that predispose carcinogenesis and poor survival.

This study also showed that coexisting pulmonary disease may exert direct effects and increase risk of mortality in men, but not in women. Because smoking status was not available in our study, although smoking is almost 10 times more prevalent in men in Taiwan,^19^ it can drive the observed differences in survival between men and women. Continued smoking after lung cancer diagnosis is associated with an increased risk of all-cause mortality and decreased survival.^35^ It has been associated with a significantly increased risk of recurrence (HR, 1.86; 95% CI, 1.01–3.41) in early-stage nonsmall cell lung cancer and development of a second primary tumor (HR, 4.31; 95% CI, 1.09–16.98).^36^ Besides, sex hormones play a role in these differences that may lead to pathogenesis of disease or serve as protective factors.^37^ Estrogen receptor-β is more frequently expressed in lung tissue in women.^38^ Estrogen receptor-β expression correlates with epidermal growth factor receptor mutations and good tumor differentiation.^39^ There were survival advantages in women and gender differences in response to epidermal growth factor receptor inhibitors and antiangiogenesis agents.^40,41^

Low serum total cholesterol concentrations increased risk of cancer deaths in British men with age-adjusted relative risk of 1.6 (95% CI, 1.1–2.3).^42^ A decreased level of preoperative high-density lipoprotein cholesterol was associated with shorter overall survival in patients with nonsmall cell lung cancer.^43^ Preoperative total serum cholesterol was significant for survival of nonsmall cell lung cancer with relative risk of 0.84 for each mmol/L increase in concentration (95% CI, 0.71–1.00).^44^ Fiorenza et al^45^ found that total cholesterol was significantly lower in patients with metastatic disease than those with nonmetastatic disease.^45^ Results from those studies are consistent with our findings.

The strengths of this study were numerous. First, our study was a retrospective cohort study with a large sample size and long follow-up. Small sample size limited reliability of previous studies and did not describe specific types of lung cancer and gender-speciﬁc analysis of risk factors. Second, our study included all stage of lung adenocarcinoma. Some studies only included surgically resected patients with lung cancer specifically with features of early-stage lung cancer. Third, there was complete ascertainment of lung cancer cell type. Fourth, to enhance the reliability of temporal relationship between pulmonary diseases and all-cause mortality of patients with lung adenocarcinoma, we included cases of asthma, COPD, and TB diagnosed 2 years before the index date because pulmonary diseases may mask symptoms and delay diagnosis of lung cancer to affect survival.

Nevertheless, this study has several limitations. First, detection bias might have been possible in patients with pulmonary diseases because of frequent hospital visits, hence, leading to a higher detection rate of early-stage lung adenocarcinoma. Second, patients with comorbid pulmonary diseases may have taken more medications that may have complicated the situation. This study did not evaluate the effects of medications. Third, NDRD, NHIRD, and TCRD do not provide detailed information about health behaviors, lifestyle information, and possible prognostic factors, such as performance status, visceral pleural invasion, and lymphovascular invasion. Such factors might have affected data analysis.

In conclusion, this study found that coexisting pulmonary diseases conferred a higher risk of mortality than any of pulmonary diseases in male patients. Because of aging and increase in prevalence of asthma, COPD, and TB, efforts to improve the survival of lung adenocarcinoma should be directed toward optimizing the management of coexisting pulmonary diseases.

## Acknowledgments

*The authors would like to thank the National Health Research Institute of Taiwan, for providing the NHIRD, and the Department of Statistics, Ministry of Health, and Welfare of Taiwan, for providing the TCRD and the NDRD. The descriptions or conclusions herein do not represent the viewpoint of the Bureau*.
